# Supplementary material for: Genome Mining, Heterologous Expression, Antibacterial and Antioxidant Activities of Lipoamides and Amicoumacins from Compost-Associated Bacillus subtilis fmb60
Source: Molecules. 2021 Mar 26;26(7):1892. doi: 10.3390/molecules26071892 (PMC8036425; doi:10.3390/molecules26071892)
Supplement: Supplementary file 1 [file molecules-26-01892-s001.pdf]

Supporting Information

Genome mining, heterologous expression, antibacterial and antioxidant activities of lipoamides and amicoumacins from compost-associated *Bacillus subtilis* fmb60

Jie Yang[1, 2, 5], Qingzheng Zhu[1], Feng Xu[1], Ming Yang[3], Hechao Du[2], Xiaoying Bian[3], Zhaoxin Lu[2], Yingjian Lu[4, *], and Fengxia Lu[2,*]

[1]School of Food Science and Engineering, Jiangsu Ocean University, Lianyungang 222005, China

[2]College of Food Science and Technology, Nanjing Agricultural University, 1 Weigang, Nanjing 210095, China

[3]Helmholtz Institute of Biotechnology, State Key Laboratory of Microbial Technology, Shandong University, Qingdao 266237, China

[4]College of Food Science and Engineering, Nanjing University of Finance and Economics, Nanjing 210003, China

[5]Jiangsu Marine Resources Development Research Institute, Lianyungang, 222000, China

*Corresponding author (Tel: +86-2584395155; Fax: +86-2584395155; E-mail: yingjianlu@nufe.edu.cn), (Tel: +86-2584395963; Fax: +86-2584395963; E-mail: lufengxia@njau.edu.cn)

Table S1. Strains and plasmids in this study

Name	Description	Ref.
E. coli strains		
GB2005	F-mcrAΔ (mrr-hsdRMS-mcrBC) φ80lacZΔM15 ΔlacX74 recA1 endA1 araD139 Δ (ara, leu) 7697 galU galK λ rpsL nupG fhuA::IS2 recET redα, phage T1-resistant	(22)
GB05-dir	GB2005, araC-BAD-ETγA	(22)
GB05-MtaA	(GB2005, mtaA-genta) a pPant transferase gene from myxobacterium *Stigmatella aurantiaca* DW4/3-1 was randomly transposed into the chromosome for expression of secondary metabolite	
GB05-MtaA-*ami*	Plasmid in *E. coli* GB05-MtaA, for expressiong of NRPS/PKS gene cluster, Cm^R	This study
GB05-MtaA-*ami*-*ace*	Plasmid in *E. coli* GB05-MtaA, for expressiong of NRPS/PKS and *ace* gene cluster, Cm^R	This study
Plasmids		
p15A-*cm*-*tet*^{R}-*ccdB*-*hyg*	PCR template to generate a linear vector for direct cloning	(23)
p15A-*cm*-*ami*	recombinant plasmid with entire *ami* gene cluster	This study
pBR322-*apra*-OriT	PCR template to generate a linear vector for *ace* gene cluster cloning	Lab stored
pBR322-*apra*-*ace*	recombinant plasmid with entire *ace* gene cluster	This study

Table S2. List of primers used in this study

Primer	Sequence (5'-3')	Description
YM-*ami*-5	GCTGTAATCTCATGTAAAGCAGGAGAAACGCCGCCCAGCTGGGTAAAAGCAACGATCGGCACAGATCCGAAAACCCCAAGTTACG	Clone *ami* gene cluster
YM-*ami*-3	CACGACTTCCGGAATGATATCGCCCGCTTTTTTGATGACGACCTTATCCAAAATCCGAATGTTTCGGCCTTGAATTGATCATATGC	Clone *ami* gene cluster
ace-pBR322-HAF	CAAATTGTCAACAGACAAGAACTAAAGAGAGTGATGCAGGTACATGAACGCTCAGTGGAACGAGGT	Clone *ace* gene cluster
ace-pBR322-HAR	CTGATTTTGCGAAAGATATCGAAACAACTGAAGGAAGCCCTGAATAACGCGTTGCTGGCGTTTTTC	Clone *ace* gene cluster
ace-F	TTATTCAGGGCTTCCTTCAGT	Clone *ace* gene cluster
ace-R	TCATGTACCTGCATCACTCT	Clone *ace* gene cluster

Table S3. Secondary Metabolite Biosynthetic Gene Clusters Identified by AntiSMASH

Clusters	Synthetase type	Most similar known cluster	Location	MIBiG BGC-ID
Cluster 1	Microcin		52718…83567	
Cluster 2	NRPS	Surfactin biosynthetic gene cluster (78% genes show similarity)	255220…320611	BGC 0000433_c1
Cluster 3	Microcin		521848…541996	
Cluster 4	NRPS/Type I PKS	xenocoumacin biosynthetic gene cluster (28% genes show similarity)	592175…673526	BGC0001054_c1
Cluster 5	Type I PKS	Bacillaene biosynthetic gene cluster (59% genes show similarity)	854869…954351	BGC0001089_c1
Cluster 6	Terpene			
Cluster 7	NRPS	Fengycin biosynthetic gene cluster (100% of genes show similarity)	1844656…1928055	BGC 0000407_c1

Cluster 8	Terpene		1995630…2017528	
Cluster 9	Type III PKS		2064193…2105290	
Cluster 10	Microcin		2914253…2934401	
Cluster 11	NRPS	Bacillibactin biosynthetic gene cluster (92% genes show similarity)	3050057…3099798	BGC 0000309_c1
Cluster 12	Thiopeptide		3117266…3146494	
Cluster 13	Sactipeptide	Subtilosin A biosynthetic gene cluster (87% of genes show similarity)	3651570…3673181	BGC0000602_c1
Cluster 14	Other	Bacilysin biosynthetic gene cluster (100% of genes show similarity)	3688160…3729578	BGC0001184_c1
Cluster 15	Microcin		4056415…4076563	
Cluster 16	Microcin		4076957…4097105	
Cluster 17	Microcin		4137409…4150267	

Table S4. Proteins encoded by *ami* cluster and open reading frames adjacent to *ami* cluster as well as their proposed function and size

Protein	Size (aa)	Proposed function	Accession No	Identity (%)
Orf 1	397	Putative MFS family major facilitator transporter (WP_003240131.1)	OEI73252.1	98
AmiA	1498	Amino acid adenylation domain-containing protein (WP_003240128.1)	OEI73253.1	96
AmiB	501	β-lactamase (WP_003240126.1)	OEI73254.1	93
AmiC	327	Hypothetical protein (WP_003240124.1)	OEI73255.1	95
AmiD	233	Thioesterase (WP_003240123.1)	OEI73256.1	97
AmiE	284	3-hydroxybutyryl-coA dehydrogenase (WP_003240121.1)	OEI73257.1	98
AmiF	353	Methoxymalonyl-ACP biosynthesis protein (WP_003240119.1)	OEI73258.1	98
AmiG	89	Acyl carrier protein (WP_003240117.1)	OEI73259.1	100
AmiH	380	Acyl-CoA dehydrogenase (NADP(+)) (WP_003240115.1)	OEI73260.1	98

AmiI	3032	Nonribosomal peptide synthetase-polyketide synthase hybrid (WP_003240114.1)	OEI73261.1	95
AmiJ	889	Nonribosomal peptide synthetase subunit (WP_003240112.1)	OEI73262.1	94
AmiK	1507	Putative polyketide synthase PksJ (PKS) (WP_003240111.1)	OEI73263.1	95
AmiL	2517	Polyketide synthase subunit (WP_003240108.1)	AFI27291.1	96
AmiM	2142	Polyketide synthase subunit (WP_003240106.1)	OEI73264.1	96
AmiN	333	Putative kinase (WP_003240104.1)	OEI73265.1	98
AmiO	459	Alkaline phosphatase (WP_003240102.1)	OEI73266.1	96
Orf 2	230	Membrane component (WP_003240098.1)	OEI73267.1	99

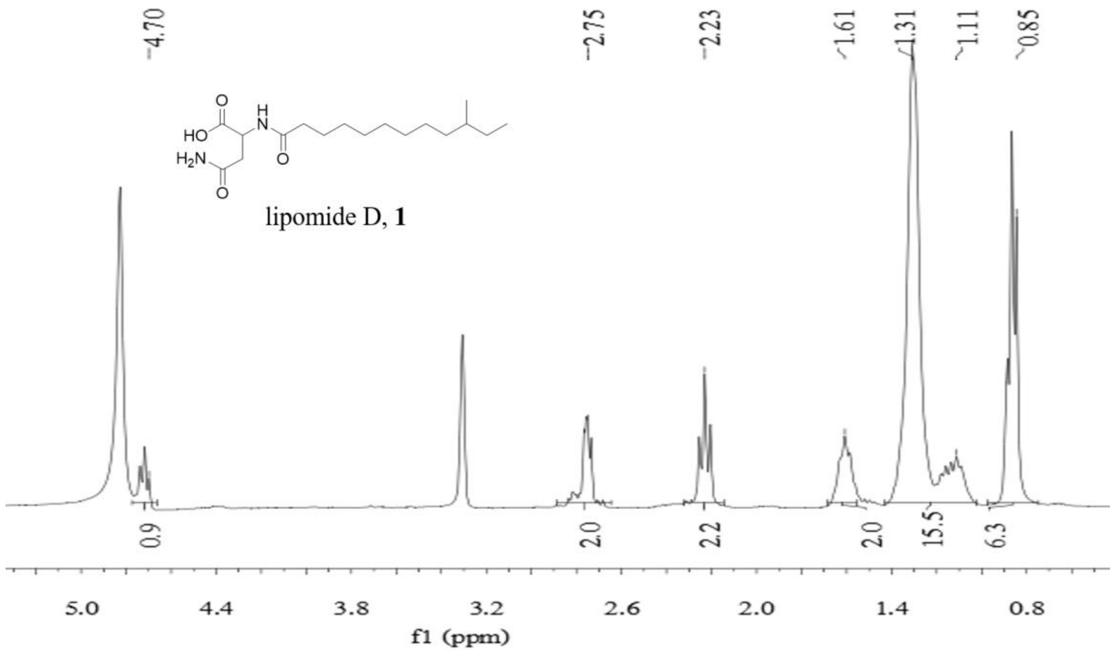

Figure S1. ^{1}H-NMR spectrum of **1** in methanol-*d*$_4$.

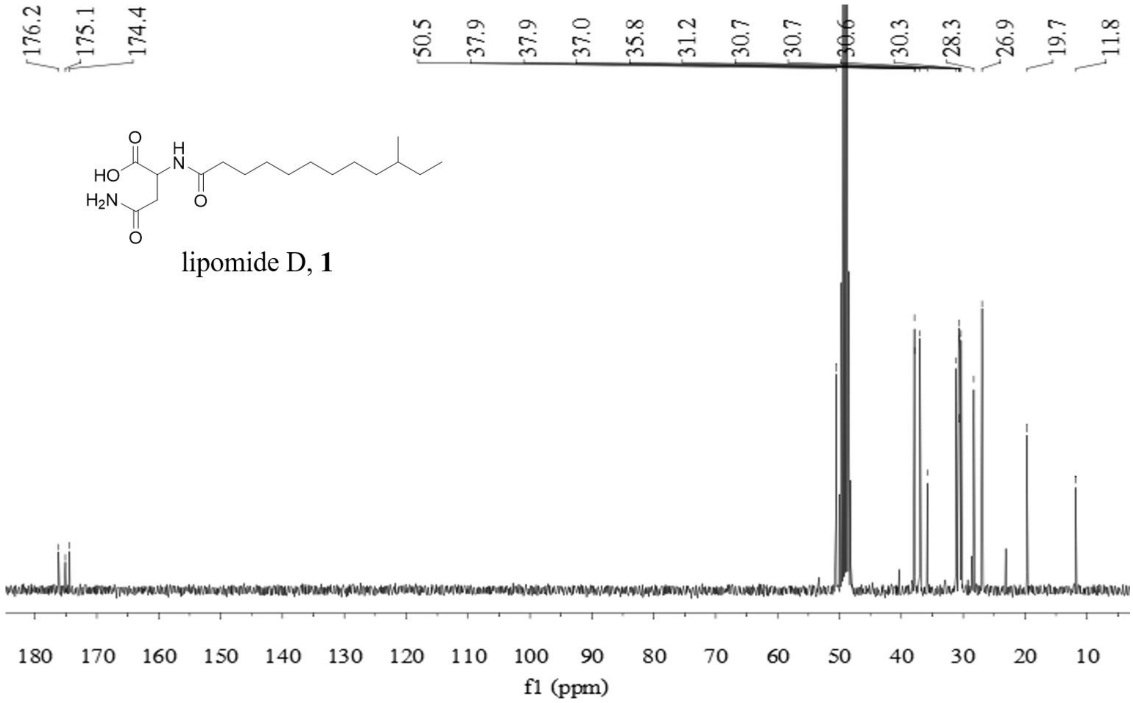

Figure S2. ^{13}C-NMR spectrum of **1** in methanol-*d*$_4$.

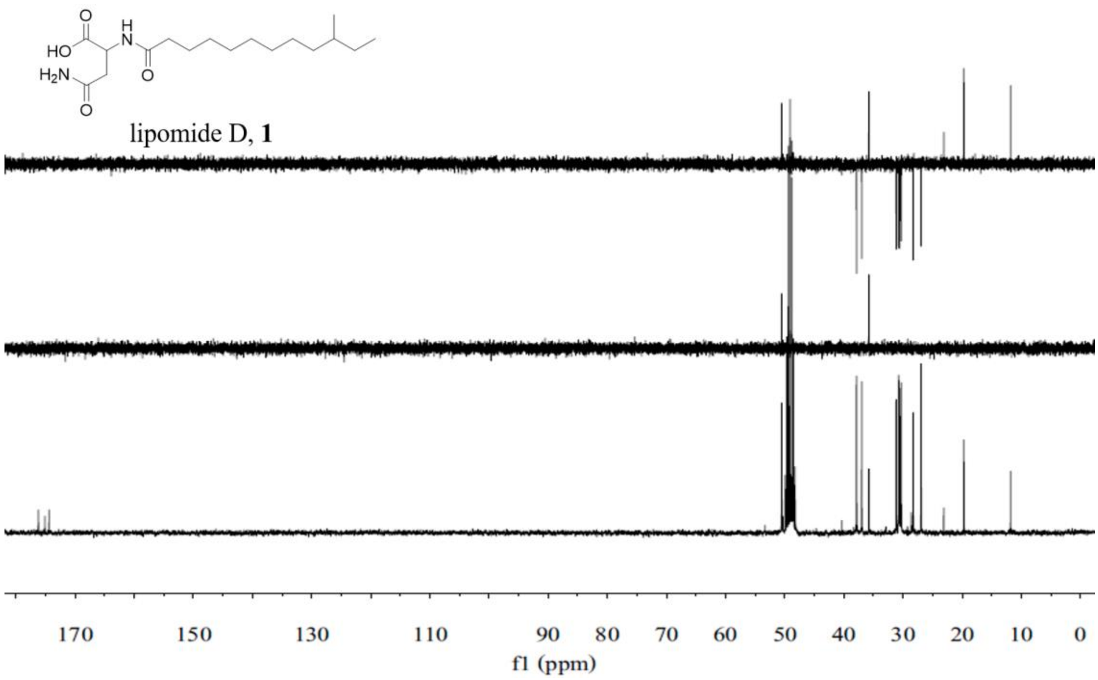

Figure S3. DEPT spectrum of **1** in methanol-d_4.

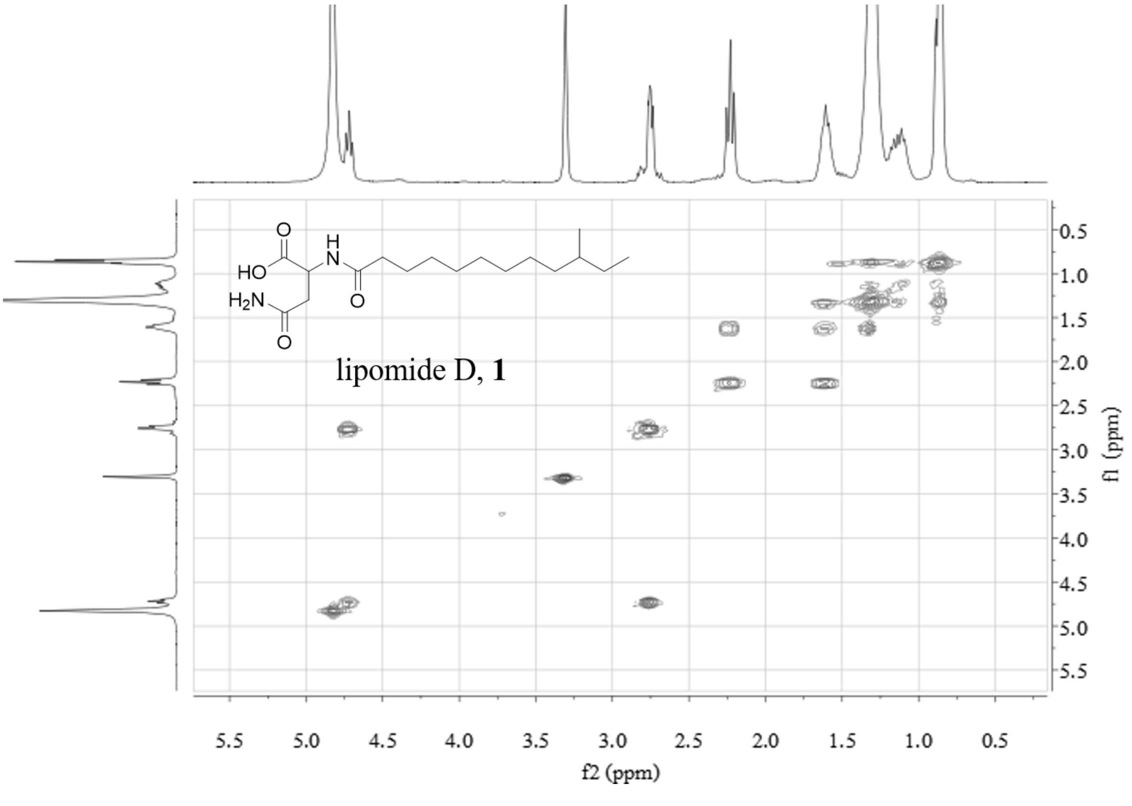

Figure S4. COSY spectrum of **1** in methanol-d_4.

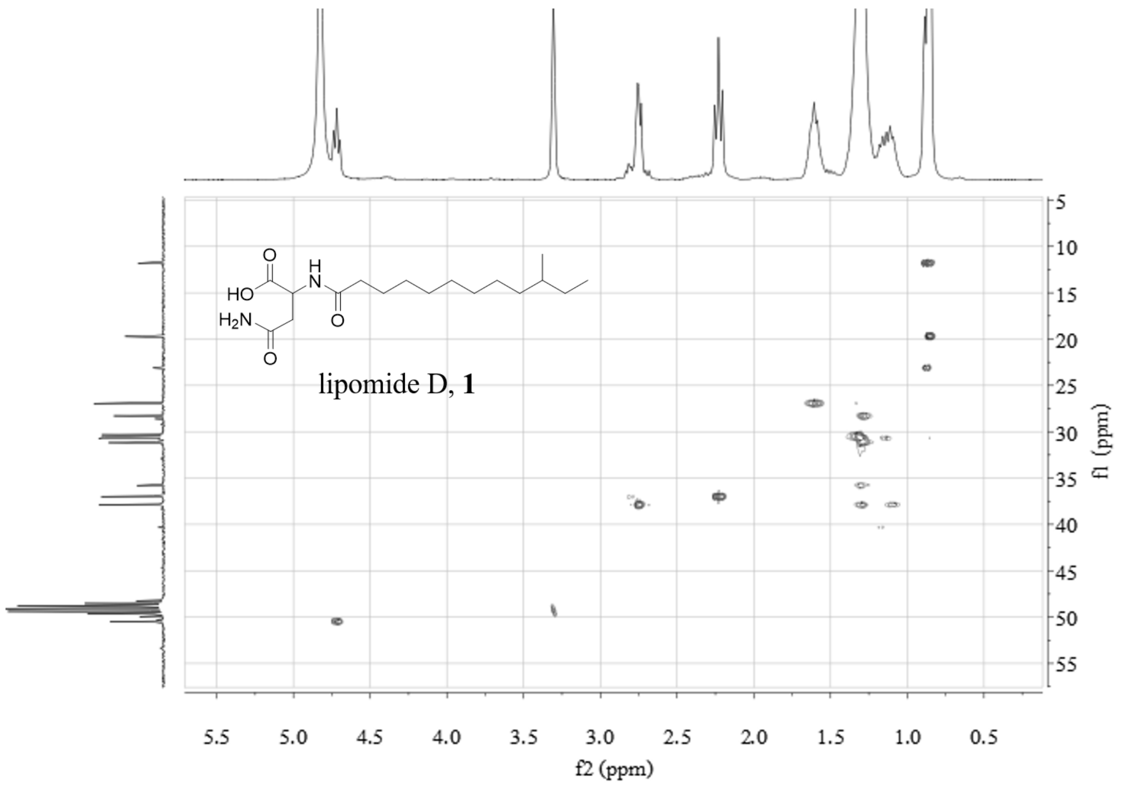

Figure S5. HSQC spectrum of **1** in methanol-d_4.

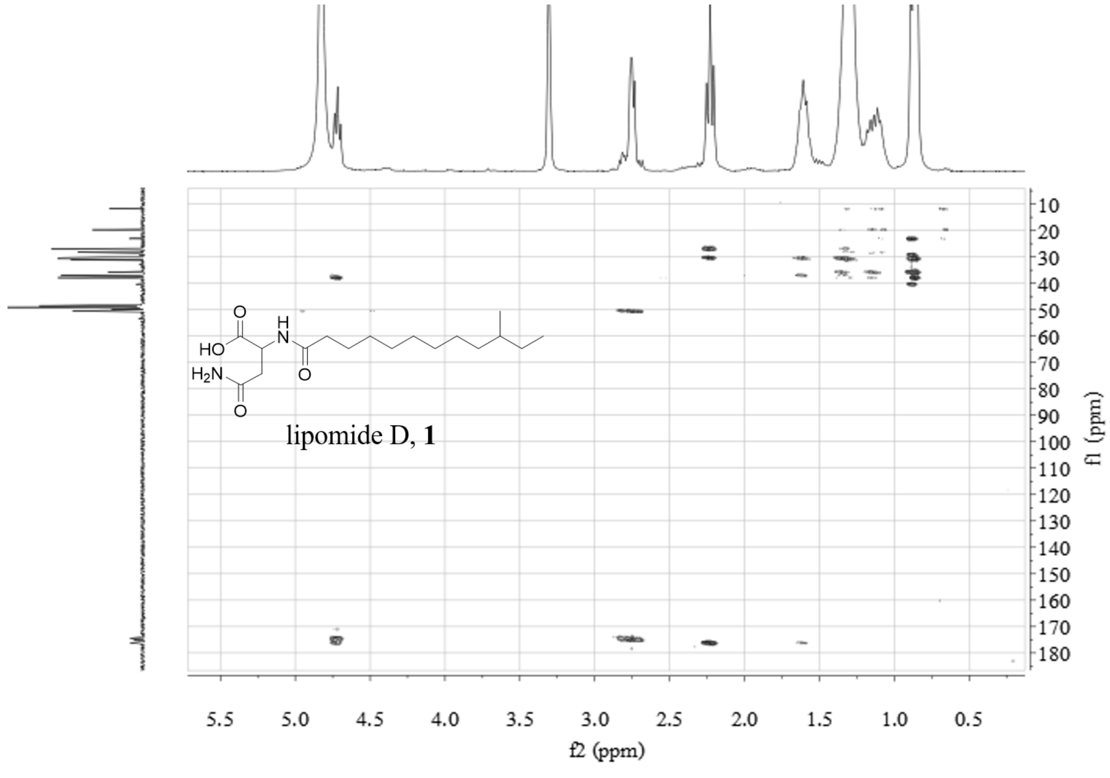

Figure S6. HMBC spectrum of **1** in methanol-d_4.

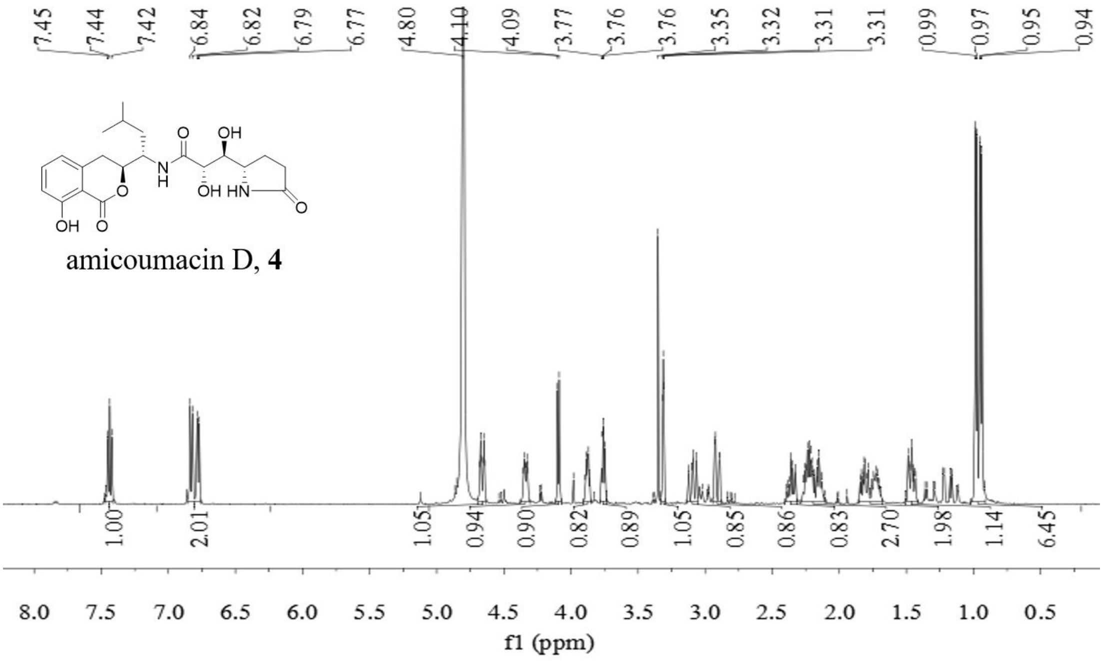

Figure S7. ^{1}H-NMR spectrum of **4** in methanol-d_4.

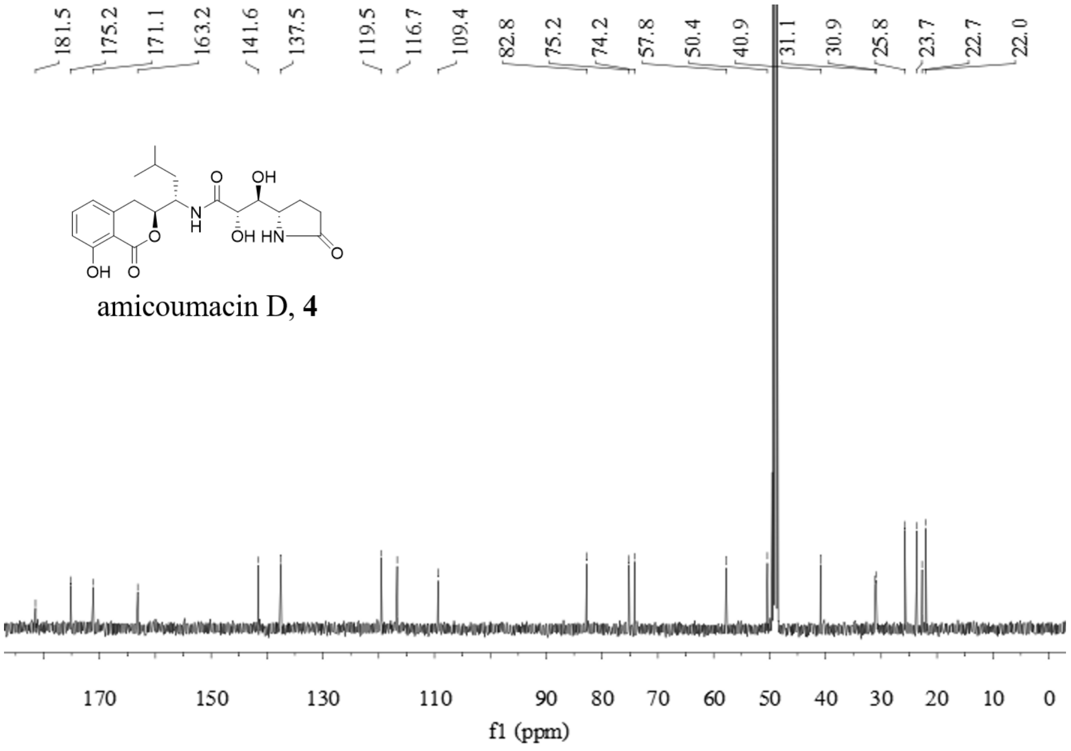

Figure S8. ^{13}C-NMR spectrum of **4** in methanol-d_4.

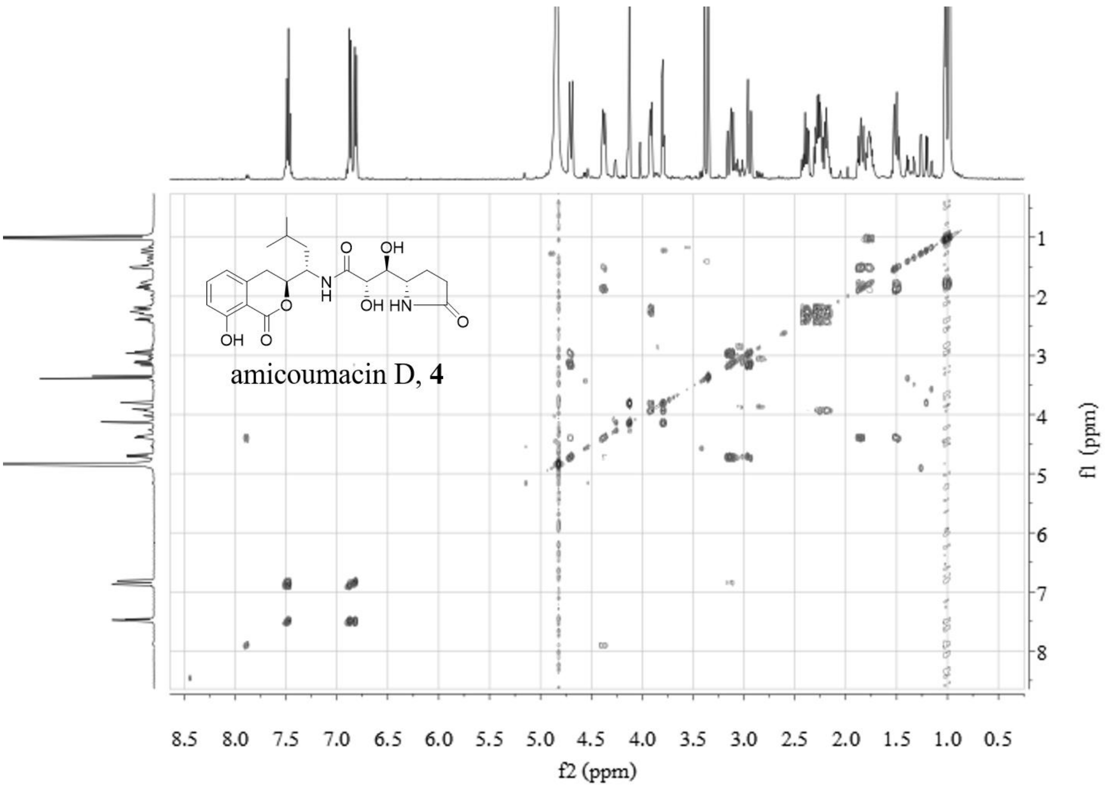

Figure S9. COSY spectrum of **4** in methanol-d_4.

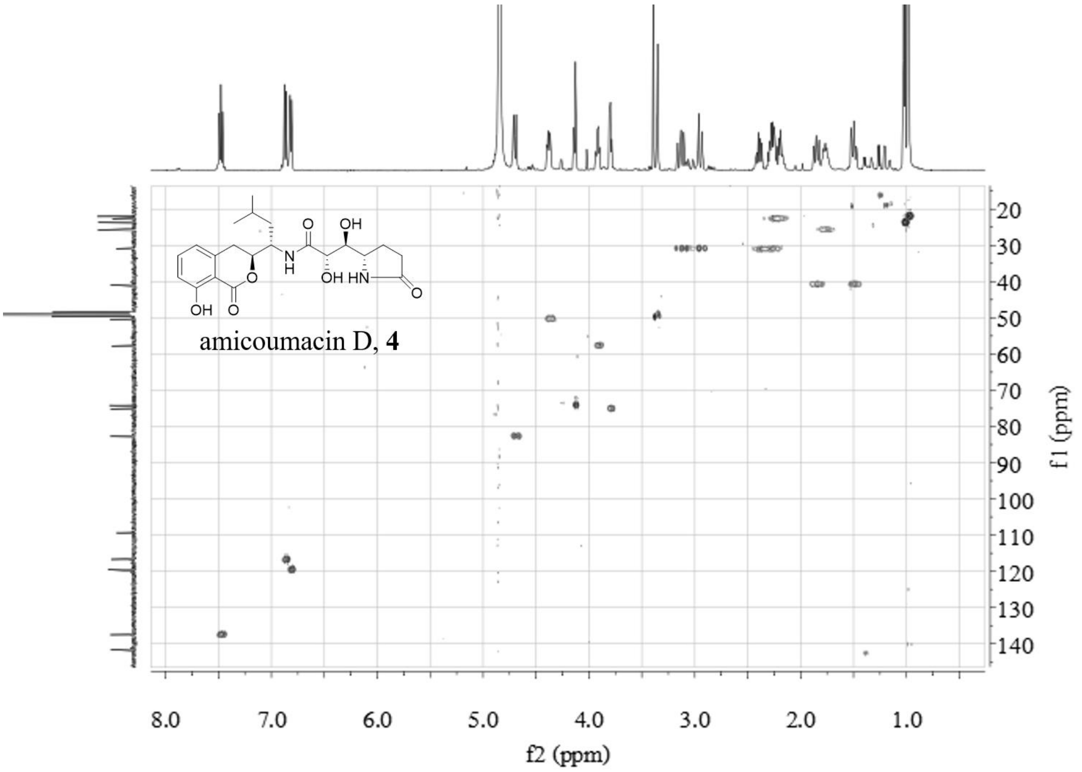

Figure S10. HSQC spectrum of **4** in methanol-d_4.

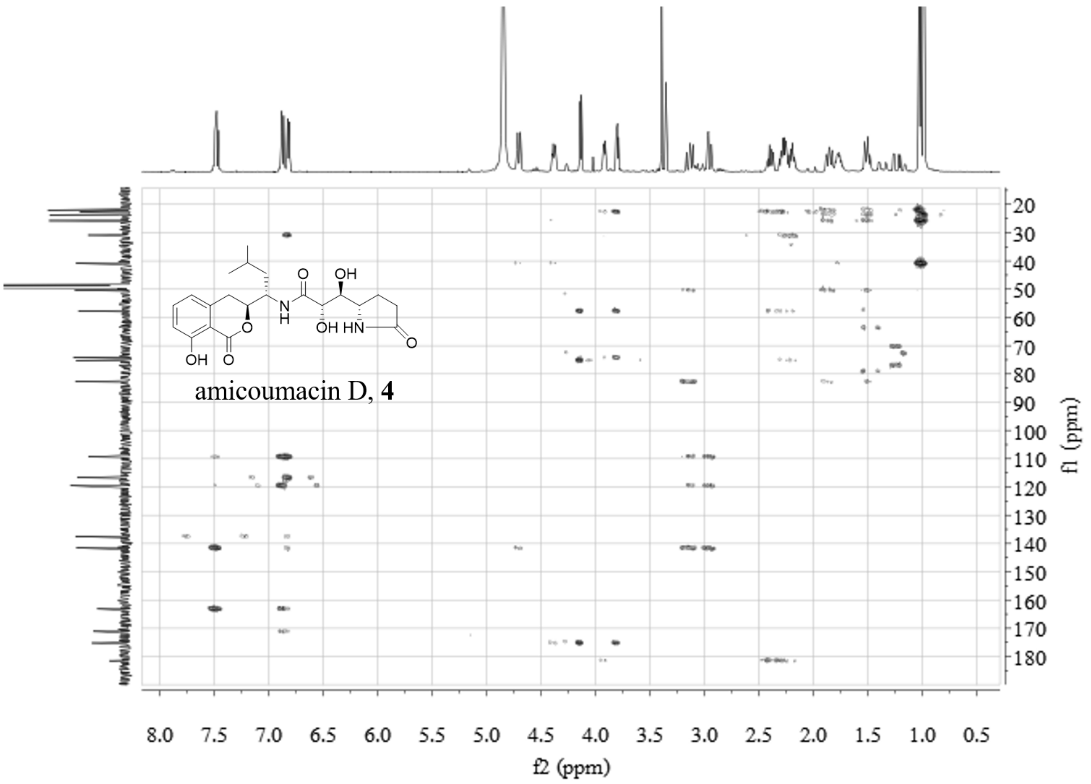

Figure S11. HMBC spectrum of **4** in methanol-d_4.

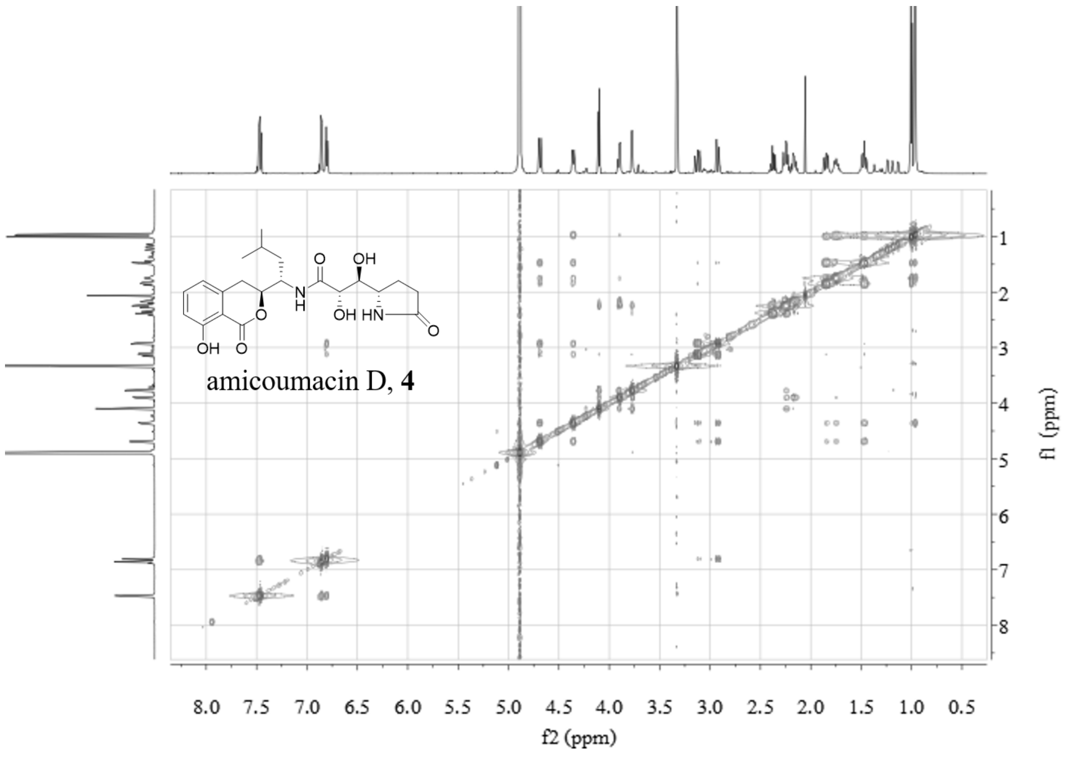

Figure S12. NOESY spectrum of **4** in methanol-d_4.

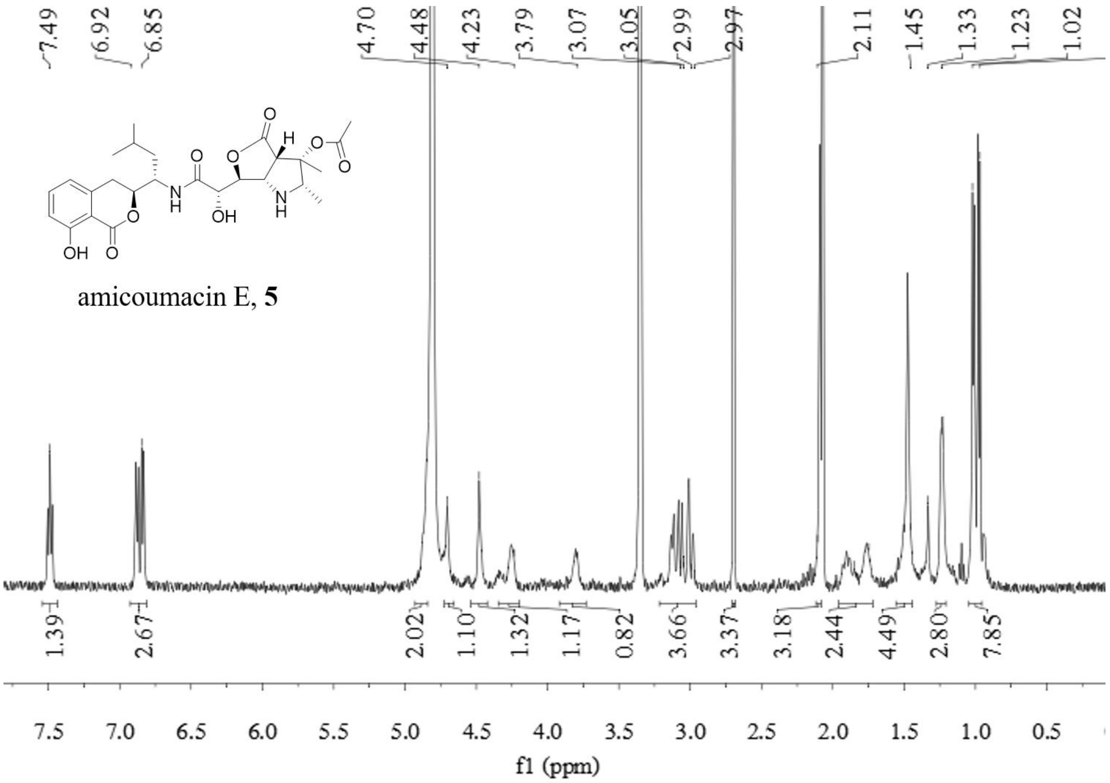

Figure S13. ^{1}H-NMR spectrum of **5** in methanol-d_4.

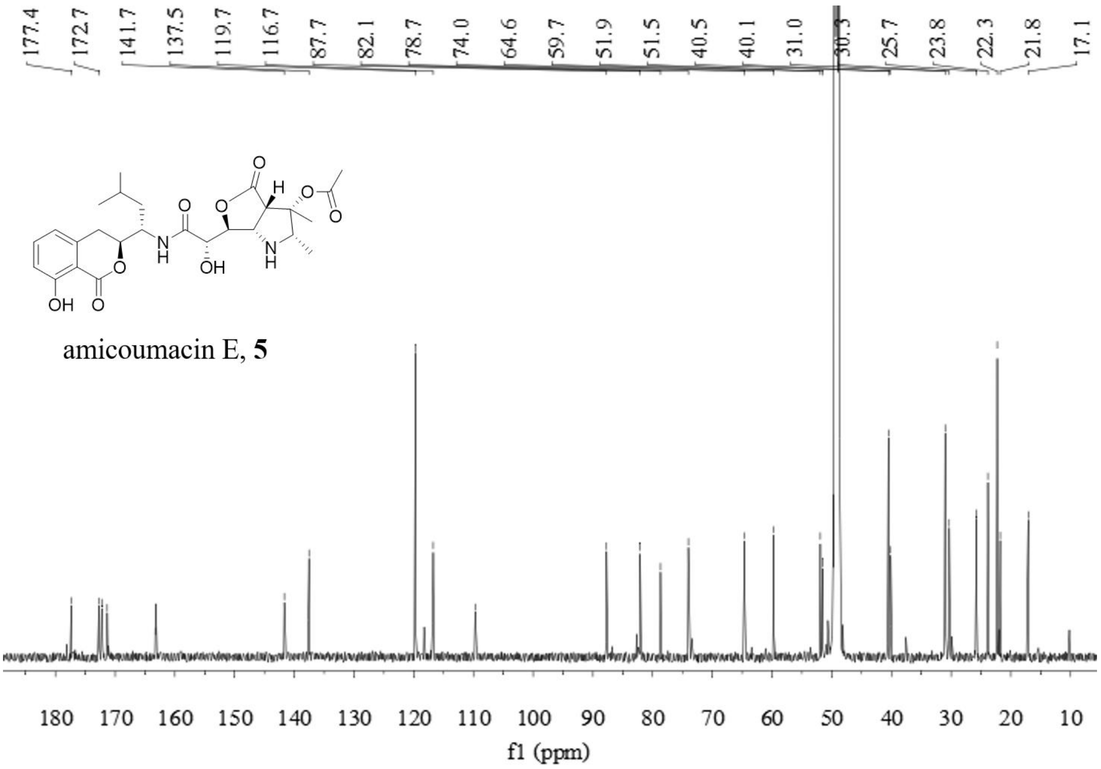

Figure 14. ^{13}C-NMR spectrum of **5** in methanol-d_4.

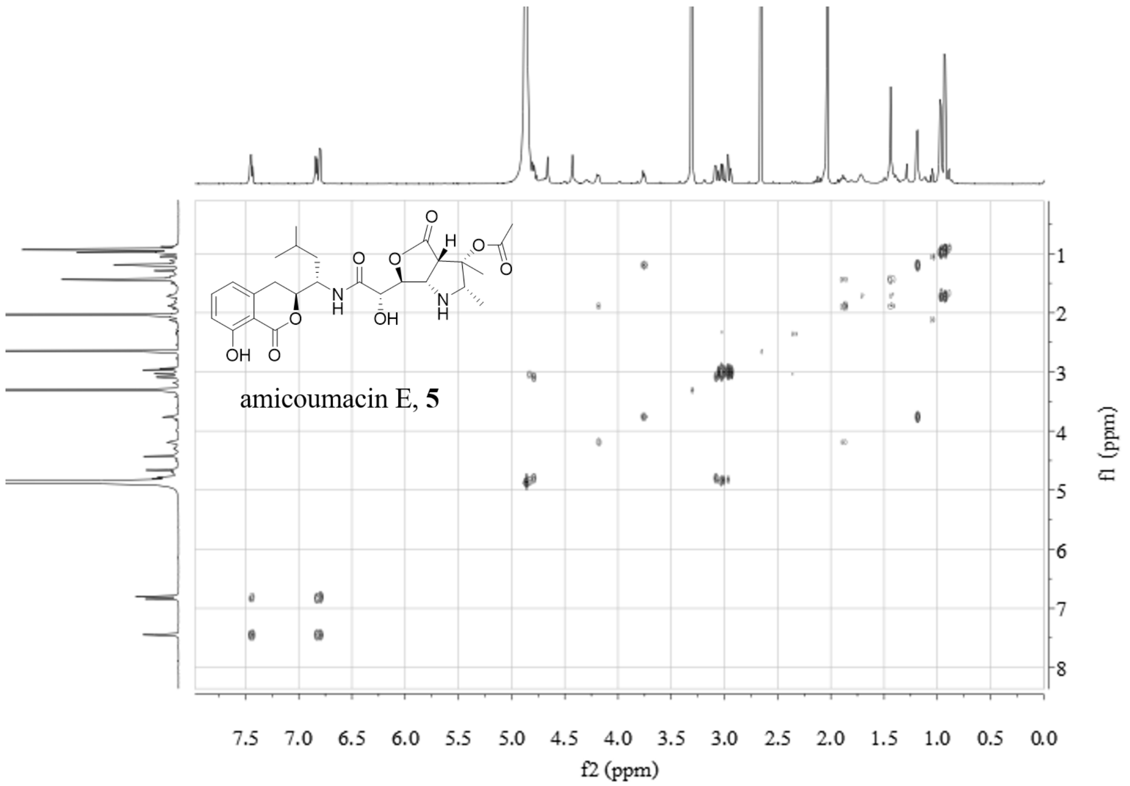

Figure S15. COSY spectrum of **5** in methanol-d_4.

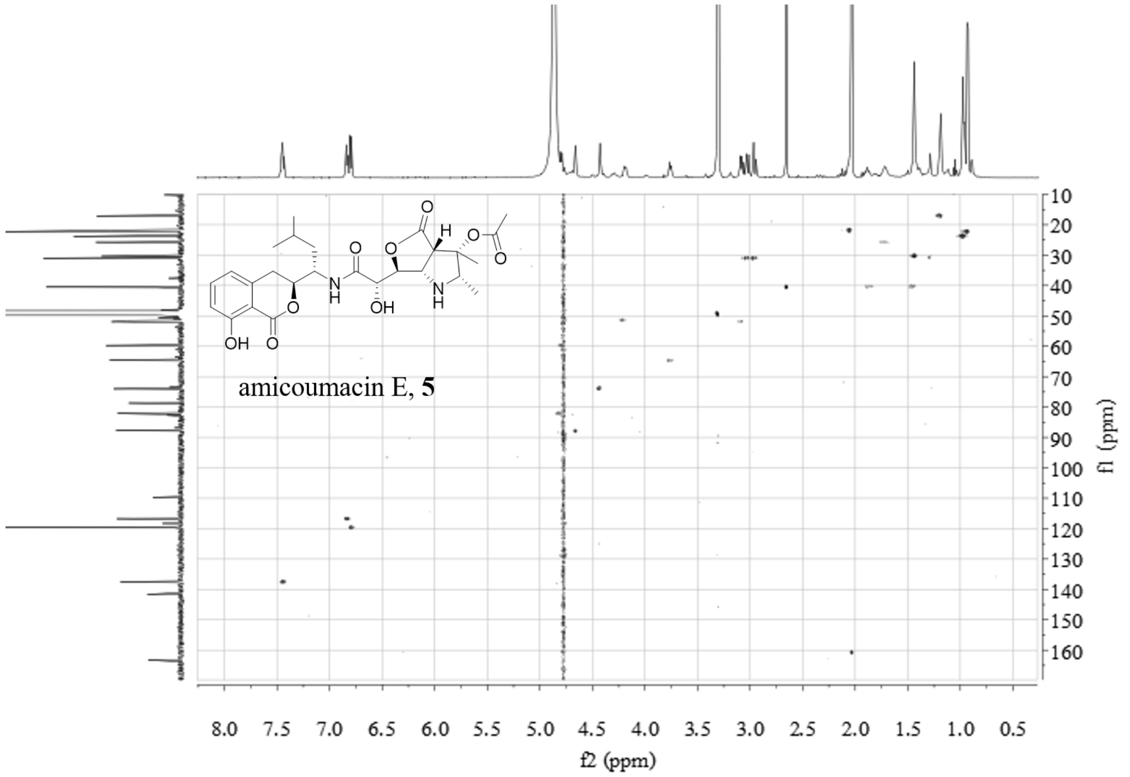

Figure S16. HSQC spectrum of **5** in methanol-d_4.

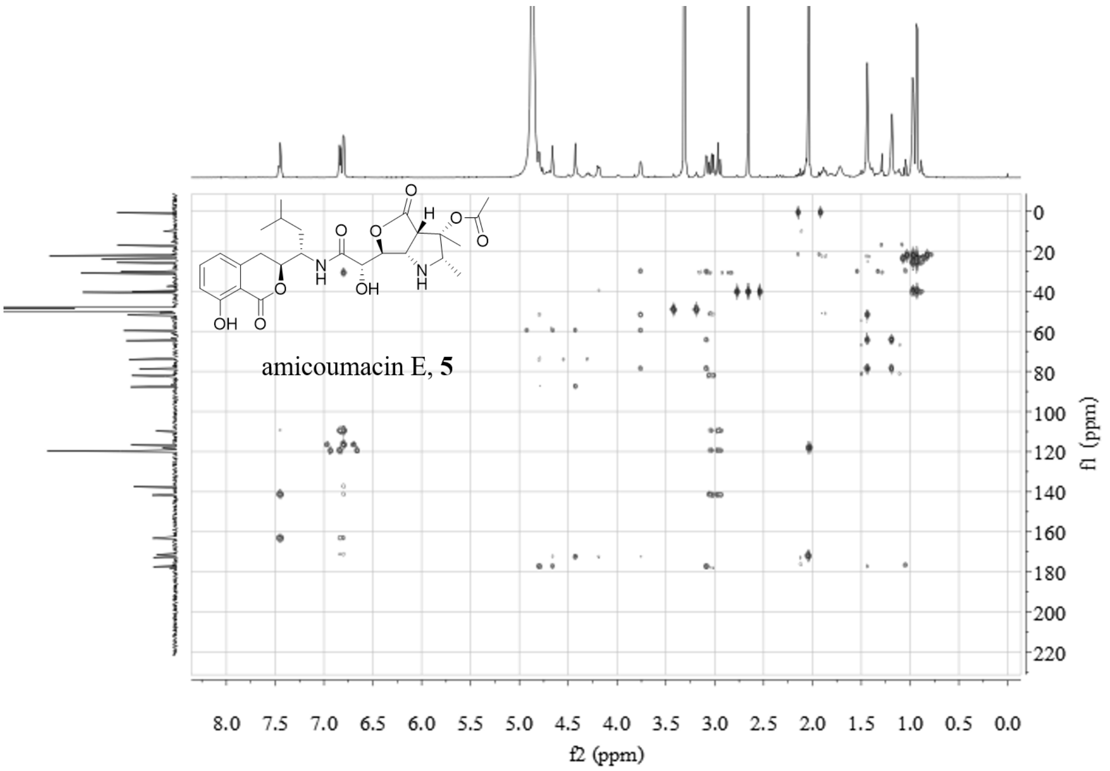

Figure S17. HMBC spectrum of **5** in methanol-d_4.

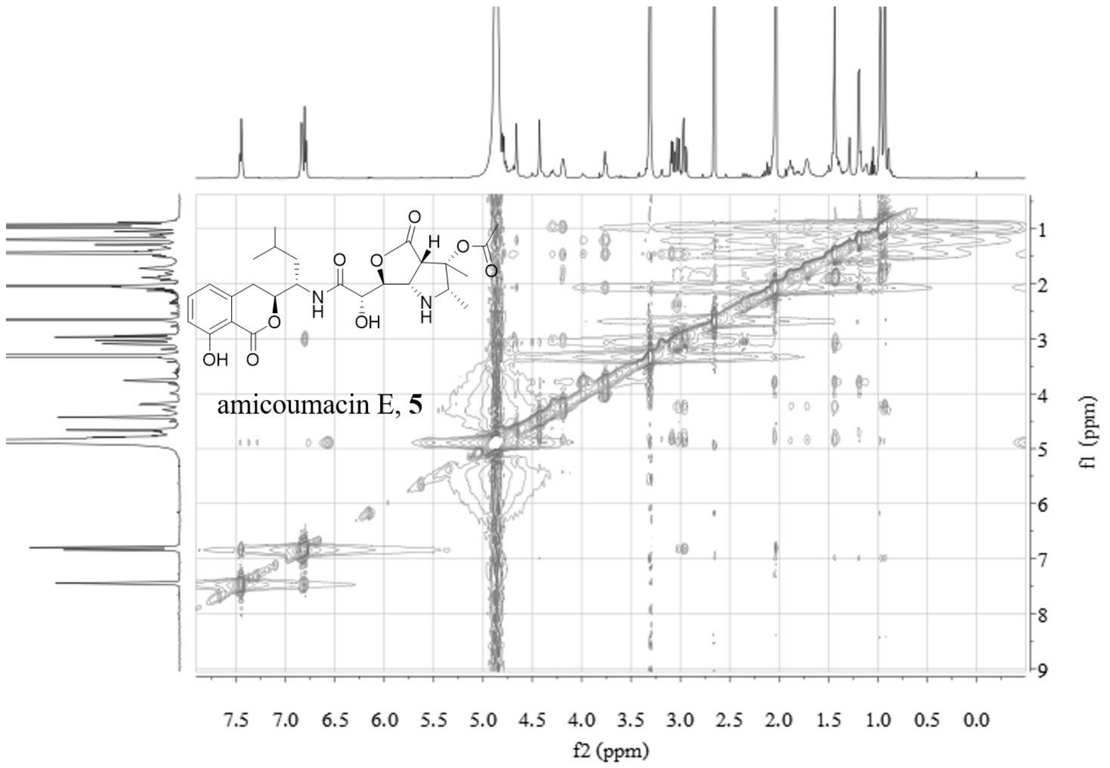

Figure S18. NOESY spectrum of **5** in methanol-d_4.

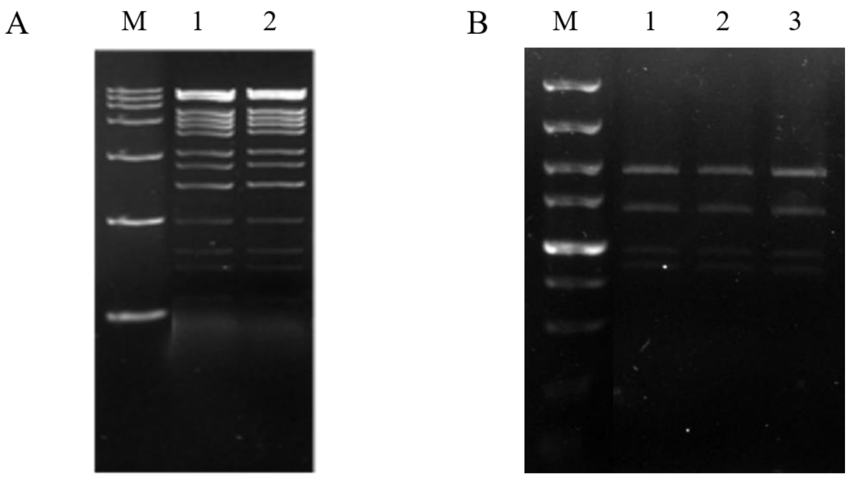

Figure S19. (A) Digestion map of NRPS/PKS gene cluster. M: DL15000 DNA Marker, 1,2: plasmid p15A-*cm-ami* digested by restriction enzyme *Afl*Ⅲ. (B) Digestion map of *ace* gene cluster. M: DL5000 DNA Marker, 1,2,3: plasmid p15A-*apra-ace* digested by restriction enzyme *Ava* I and *Eco* RV.
